# Ciprofloxacin Enhances the Chemosensitivity of Cancer Cells to ABCB1 Substrates

**DOI:** 10.3390/ijms20020268

**Published:** 2019-01-11

**Authors:** Pranav Gupta, Hai-Ling Gao, Yunali V. Ashar, Nishant M. Karadkhelkar, Sabesan Yoganathan, Zhe-Sheng Chen

**Affiliations:** 1 Department of Pharmaceutical Sciences, College of Pharmacy and Health Sciences, St. John’s University, Queens, NY 11439, USA; pgupta0@mgh.harvard.edu (P.G.); gaohailing1227@163.com (H.-L.G.); yunali.ashar17@my.stjohns.edu (Y.V.A.); nishant.karadkhelkar14@my.stjohns.edu (N.M.K.); yoganats@stjohns.edu (S.Y.); 2 Department of Histology and Embryology, Weifang Medical University, Weifang 261053, China

**Keywords:** ciprofloxacin, ABCB1 transporter, paclitaxel, multidrug-resistance

## Abstract

ABCB1 is one of the major drug efflux transporters that is known to cause multidrug resistance (MDR) in cancer patients receiving chemotherapy for the treatment of solid tumors and hematological malignancies. Inhibition of ABCB1 efflux function is important for maintaining the intracellular concentration of chemotherapeutic drugs. Here, we evaluated ciprofloxacin for its ability to reverse MDR caused by the overexpression of ABCB1. Cytotoxicity of ciprofloxacin was determined by the MTT assay. The chemosensitizing effects of ciprofloxacin were determined in combination with ABCB1 substrates. The intracellular accumulation and efflux of ABCB1 substrates was measured by a scintillation counter, and protein expression was determined by the Western blotting. Vanadate-sensitive ATPase assay was performed to determine the effect of ciprofloxacin on the ATPase activity of ABCB1, and docking analysis was done to determine the interaction of ciprofloxacin with ABCB1. Ciprofloxacin significantly potentiated the cytotoxic effects of ABCB1 substrates in ABCB1-overexpressing cells. Furthermore, ciprofloxacin increased the intracellular accumulation and decreased the efflux of [^3^H]-paclitaxel without altering the expression of ABCB1. Ciprofloxacin stimulated the ATPase activity of ABCB1 in a concentration-dependent manner. Our findings showed that ciprofloxacin potently inhibits the ABCB1 efflux function and it has potential to be developed as a combination anticancer therapy.

## 1. Introduction

Chemotherapy remains the most successful and reliable treatment option for combating hematological malignancies and solid tumors [1,2,3]. The chronic use of chemotherapeutic drugs renders cancer cells insensitive, thus, requiring a higher dose of the drug to exert the same pharmacological effect. This phenomenon of the development of insensitivity in cancer cells to a wide variety of chemotherapeutic drugs that have different structures and mechanisms of action is referred to as multidrug resistance (MDR) [4,5,6].

The development of MDR in cancer cells is known to be the major factor hindering the success of chemotherapy in clinics [7,8,9]. With the advancement in translational medicine and system therapeutics, a number of intrinsic and extrinsic mechanisms have been identified to cause MDR in cancer cells. However, the ATP-binding cassette (ABC) transporters-mediated efflux of chemotherapeutic drugs remains the most prominent mechanism causing MDR [10,11]. The ABC transporter family is one of the largest known protein families consisting of 49 members, divided into seven subfamilies (A through G) [12,13,14]. The first member of the ABCB family, ABCB1, also known as P-glycoprotein (P-gp), is the most extensively studied ABC transporter to induce MDR in cancer cells [12,15,16]. ABCB1, a 170-kDa protein, is an apical membrane transporter located in the kidney, placenta, intestine, and brain where it exerts cytoprotective effects by effluxing toxins and reducing their accumulation in these vital organs [1,8,11]. However, in cancer cells, the overexpression of ABCB1 may cause a decrease in the intracellular accumulation of chemotherapeutic drugs, causing MDR. ABCB1 effluxes a number of substrate chemotherapeutic drugs such as paclitaxel, doxorubicin, vincristine, vinblastine, and colchicine [17,18].

It is of great importance to develop novel inhibitors that could target the efflux function of ABC transporters and overcome MDR. A number of small molecule inhibitors such as imatinib, nilotinib, and erlotinib have been reported to increase the intracellular accumulation of substrate drugs and reverse ABCB1-mediated MDR [1,4,5,8,11,12,13,17]. Recently, a number of studies have reported that antibiotics could reverse ABC transporters-mediated MDR [19,20,21,22,23]. In the light of such evidences, we investigated if ciprofloxacin could reverse ABCB1-mediated MDR.

Ciprofloxacin belongs to the second generation of fluoroquinolone antibiotics that exhibits potent antimicrobial activity and is used for the treatment of various bacterial infections [24,25]. Studies conducted in the parental MDCKI and ABCB1 overexpressing MDCKI-MDR1 cells have found ciprofloxacin to be a substrate of ABCB1 [26]. However, studies conducted on the parental LLC-PK1 and ABCB1 overexpressing L-MDR1 cells found ciprofloxacin to be an inhibitor of ABCB1 [26,27]. Thus, it is pertinent to confirm ciprofloxacin’s inhibitory or substrate function in regards to ABCB1.

## 2. Results

### 2.1. Cytotoxic Effects of Ciprofloxacin on Parental and ABCB1-Overexpressing Cells

**Figure 1:**
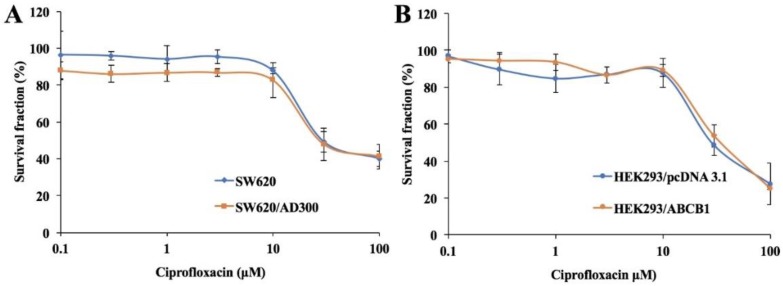
Cytotoxicity of ciprofloxacin in parental and drug-resistant cells. Cell survival (%) percentage was measured after treatment with ciprofloxacin for 72 h in parental and drug-resistant cells: SW620 and SW620/AD300 cells (**A**), HEK293/pcDNA3.1 and HEK293/ABCB1 cells (**B**). Points with error bars represent the mean ±SD for independent determinations in triplicate. The above figures are representative of three independent experiments.

In order to determine the non-toxic concentration of ciprofloxacin, its cytotoxicity was evaluated using the MTT assay. As shown in Figure 1A,B, more than 85% of both parental and ABCB1-overexpressing cells survived at the concentration of 10 μM ciprofloxacin. These results indicated that ciprofloxacin is safe to be used up to a concentration of 10 μM. Thus, ciprofloxacin at 1, 5, and 10 μM was tested in combination with chemotherapeutic substrates of ABCB1 for its ability to reverse ABCB1-mediated MDR.

### 2.2. Effect of Ciprofloxacin on the Drug Sensitivity of ABCB1-Overexpressing Cells

To investigate the reversal effects of ciprofloxacin on ABCB1-overexpresing cells, cell survival assays were performed in the presence and absence of ciprofloxacin. Paclitaxel and vincristine were used as ABCB1 substrates and cisplatin was used as a non-ABCB1 substrate. The ABCB1-overexpressing SW620/AD300 cells exhibited resistance to ABCB1 substrates, such as paclitaxel and vincristine, compared with parental SW620 cells (Table 1). Ciprofloxacin at 1, 5, and 10 μM significantly sensitized the SW620/AD300 cells to the substrates of ABCB1. Similarly, ciprofloxacin at 1, 5, and 10 μM sensitized the transfected HEK293/ABCB1 cells to the substrates of ABCB1 (Table 2). Verapamil at 10 μM was used as a positive control inhibitor of ABCB1.

### 2.3. Effect of Ciprofloxacin on the Intracellular Accumulation of [^3^H]-Paclitaxel

**Figure 2:**
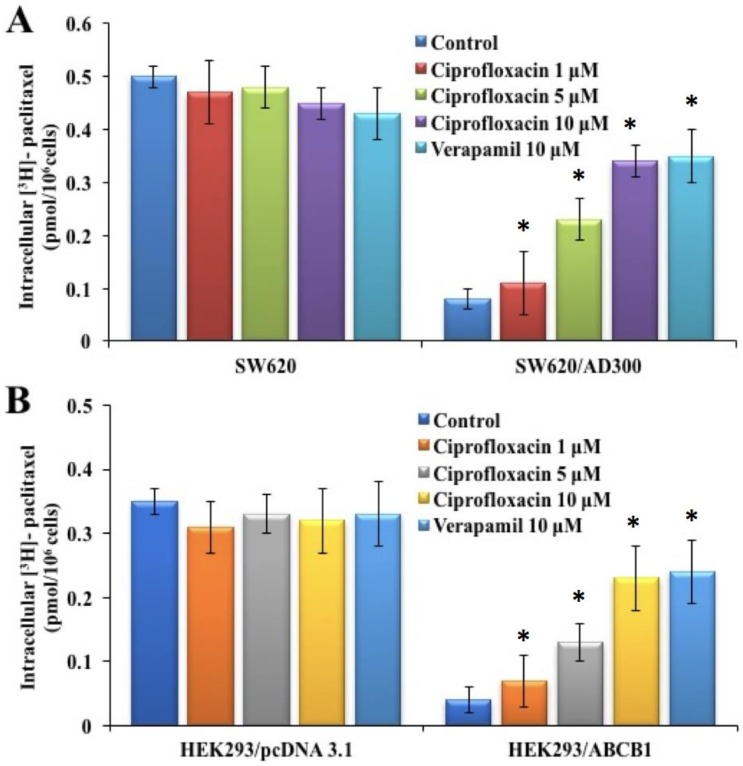
Effect of ciprofloxacin on the accumulation of [^3^H]-paclitaxel. The effect of ciprofloxacin on accumulation of [^3^H]-paclitaxel in SW620 and SW620/AD300 cells (**A**) and HEK293/pcDNA3.1 and HEK293/ABCB1 cells (**B**). Columns are the mean of triplicate determinations; the error bars represent the SD, * *p* < 0.05 versus the control group. Verapamil 10 μM is used as positive control for ABCB1-overexpressing cells.

In order to determine the mechanism by which ciprofloxacin attenuates ABCB1-mediated MDR, we investigated the effect of ciprofloxacin on the intracellular accumulation of [^3^H]-paclitaxel in parental SW620 and HEK293/pcDNA3.1 and ABCB1-overexpressing SW620/AD300 and HEK293/ABCB1 cells. The intracellular accumulation of [^3^H]-paclitaxel in SW620/AD300 and HEK293/ABCB1 cells was significantly lower than those in SW620 and HEK293/pcDNA3.1 cells, after incubation with [^3^H]-paclitaxel for 2  h (Figure 2). Treatment with ciprofloxacin significantly increased the intracellular accumulation of [^3^H]-paclitaxel in SW620/AD300 and HEK293/ABCB1 cells, in a concentration-dependent manner. In addition, ciprofloxacin at 1, 5, and 10 µM had no effect on the intracellular accumulation levels of [^3^H]-paclitaxel in SW620 and HEK293/pcDNA3.1 cells (Figure 2A,B), indicating that its action is specific to ABCB1 efflux function. The effects obtained from ciprofloxacin treatment were comparable to the intracellular accumulation of [^3^H]-paclitaxel achieved by treatment with verapamil at 10 µM.

### 2.4. Effect of Ciprofloxacin on the Efflux of [^3^H]-Paclitaxel

**Figure 3:**
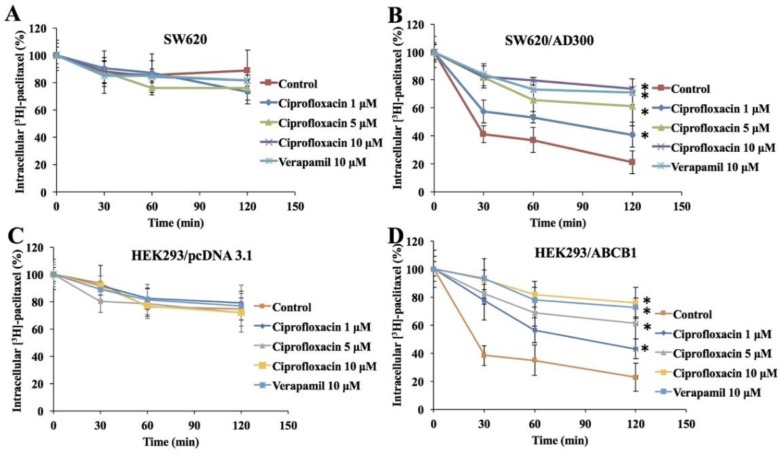
Effect of ciprofloxacin on the efflux of the substrates of ABCB1. A time course (0, 30, 60, 120 min) versus the percentage of intracellular [^3^H]-paclitaxel remaining (%) was plotted to show the effect of ciprofloxacin in SW620 (**A**), SW620/AD300 (B), HEK293/pcDNA3.1 (**C**), and HEK293/ABCB1 (**D**) cells. Error bars represent the SD, *p < 0.05 versus the control group. Verapamil 10 μM is used as positive control for ABCB1-overexpressing cells.

To confirm that the increase in intracellular accumulation of [^3^H]-paclitaxel was due to the inhibition of ABCB1 efflux function, we determined the efflux function of [^3^H]-paclitaxel from parental and ABCB1-overexpressing cells. As shown in Figure 3, in the absence of ciprofloxacin, the remaining intracellular amount of [^3^H]-paclitaxel in SW620/AD300 and HEK293/ABCB1 cells was significantly lower than that of HEK293/pcDNA3.1 and SW620 cells, due to the efflux of [^3^H]-paclitaxel by ABCB1. Treatment with ciprofloxacin (1, 5, and 10 µM) significantly decreased the efflux of [^3^H]-paclitaxel from SW620/AD300 and HEK293/ABCB1 cells in a time-dependent manner. Moreover, these results were comparable to the decrease in efflux of [^3^H]-paclitaxel by verapamil at 10 µM.

### 2.5. Effect of Ciprofloxacin on the Expression of ABCB1

**Figure 4:**
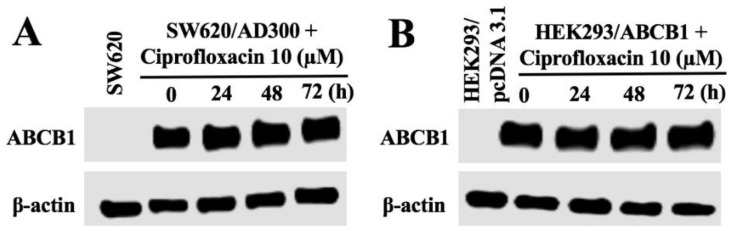
Effect of ciprofloxacin on the expression of ABCB1. The effect of ciprofloxacin on the expression of ABCB1 was tested after the cells were treated with 10 μM ciprofloxacin for 0, 24, 48, 72, and 96 h (**A**,**B**). Equal amounts of total cell lysates were used for each sample and a Western blot analysis was performed.

Since the reversal effect of ciprofloxacin could be either due to the blockade of the efflux function or a decrease in the expression of ABCB1 transporter, we conducted the Western blot analysis to determine the effect of ciprofloxacin on the expression of ABCB1. As shown in Figure 4, upon treatment with ciprofloxacin at 10 µM, there was no significant change in the protein expression of ABCB1 in SW620/AD300 and HEK293/ABCB1 cells. These results indicated that the sensitization effect of ciprofloxacin did not result from the alteration of ABCB1 expression.

### 2.6. Effect of Ciprofloxacin on the ATPase Activity of ABCB1

**Figure 5:**
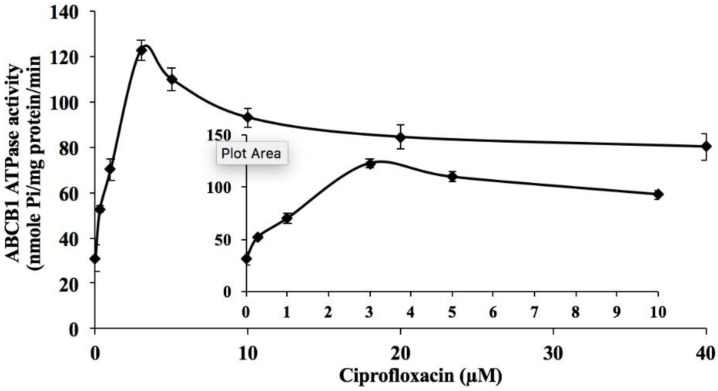
Effect of ciprofloxacin on orthovanadate (Vi)-sensitive ABCB1 ATPase activity. Crude membranes (10 μg protein/reaction) from High-five cells expressing ABCB1 were incubated with increasing concentrations of ciprofloxacin (0–40 μM). The inset shows stimulation of ATP hydrolysis at concentration of 0–10 μM ciprofloxacin. The mean values are plotted and error bars depict SD.

To determine the effect of ciprofloxacin on the ATPase activity of ABCB1, we measured ABCB1-mediated ATP hydrolysis in the presence of ciprofloxacin (0–40 µM). As shown in Figure 5, ciprofloxacin stimulated the vanadate-sensitive ATPase activity of ABCB1 in a concentration-dependent manner with a fold-stimulation of 3.9-fold of the basal activity.

### 2.7. Docking

**Figure 6:**
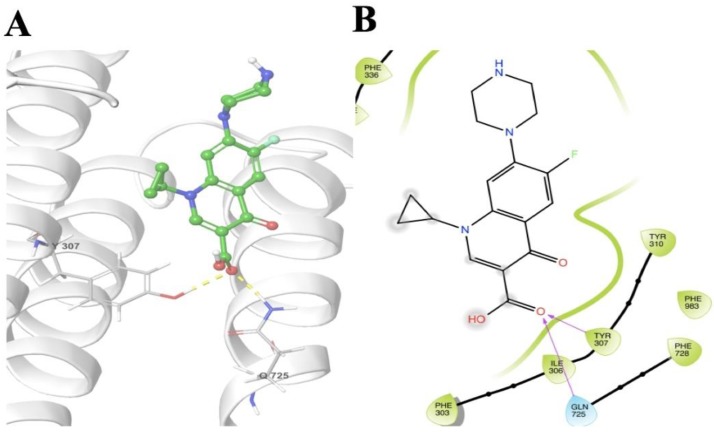
Molecular interaction of ciprofloxacin with the human homology ABCB1. Extra precision docking model of ciprofloxacin within transmembrane domains of homology modeled human ABCB1. (**A**) Docking pose of ciprofloxacin. Amino acid residues are illustrated as thin tubes with the color representations as follows: carbon in gray, hydrogen in white, nitrogen in blue, oxygen in red, and sulfur in yellow. The protein is represented in white colored ribbon form. The inhibitor is represented by the ball and stick model with identical colors as above except that carbon atoms are represented in green. Blue dashes represent the π-π stacking interaction, yellow dashes represent the hydrogen bonding and the salt bridge is indicated by purple dashes. (**B**) 2-D representation of docking pose of ciprofloxacin. The colored drops (green indicate hydrophobic) indicate amino acid residues within 5 Å of the ligand and the arrows (magenta) show hydrogen bonds.

The molecular interactions of ciprofloxacin with homology modeled human ABCB1/P-gp showed interactions within the transmembrane domains with a docking score of −6.152 kcal mol^−1^ (Figure 6). Tyr307 showed hydrogen bonding between its side chain phenolic hydrogen and the carbonyl oxygen of ciprofloxacin. There is one more hydrogen bond observed between the side chain amide hydrogen of Gln725 and the carbonyl oxygen of ciprofloxacin.

## 3. Discussion

Ciprofloxacin has been extensively studied for its anticancer effects both in vitro and in vivo. Studies have shown that ciprofloxacin inhibits the cellular proliferation of melanoma cells B16F10, triple-negative breast cancer cells MDA-MB−231, pancreatic cancer cells Panc−1, non-small cell lung cancer cells A549, and hepatocellular carcinoma cells HepG2 [28,29,30,31]. Here, we report the chemosensitizing effects of ciprofloxacin by inhibiting the efflux function of ABCB1 transporter.

One major finding of this study was that ciprofloxacin (1, 5, and 10 μM), in a concentration-dependent manner, significantly sensitized the ABCB1-overexpressing cells to the substrates of ABCB1. As evident from our MTT results, ciprofloxacin produced a significant decrease in the IC_50_ values of paclitaxel and vincristine in ABCB1-overexpressing cells. This effect was comparable to verapamil, a well-known inhibitor of ABCB1 efflux function. Furthermore, ciprofloxacin did not enhance the cytotoxicity of cisplatin, a drug that is not a substrate for the ABCB1 transporter. These results suggested that ciprofloxacin is specific in its action to the substrates of the ABCB1 transporter.

In order to understand the mechanism by which ciprofloxacin sensitizes the ABCB1-overexpressing cells to the substrates of ABCB1, we assessed the effects of ciprofloxacin on the intracellular accumulation and efflux of [^3^H]-paclitaxel. Consistent with previous studies, our findings indicated that ciprofloxacin produces a significant concentration-dependent increase in the intracellular accumulation of [^3^H]-paclitaxel in cells expressing ABCB1 [32,33,34]. Furthermore, ciprofloxacin produces a significant concentration-dependent decrease in the efflux of [^3^H]-paclitaxel from cells overexpressing the ABCB1 transporter. This effect was comparable to verapamil at 10 μM. These results suggested that ciprofloxacin increases the sensitivity of ABCB1-overexpressing cells to paclitaxel by inhibiting its efflux from the cells.

Since it could be argued that the chemosensitizing effects of ciprofloxacin may result from a decrease in the expression of ABCB1, we performed Western blotting to assess the effect of ciprofloxacin on the expression of ABCB1, in cells overexpressing the ABCB1 transporter. Our results clearly indicated that ciprofloxacin (10 μM) did not alter the expression of ABCB1 upon treatment for 72 h, thus, indicating that the sensitizing effects of ciprofloxacin is directly related to its inhibition of the function of the ABCB1 transporter. It is well known that the ABC transporters efflux substrate drugs by utilizing the energy derived from the hydrolysis of ATP by the enzyme ATPase and liberation of inorganic phosphate (pi) [7,11,35,36]. We assessed whether ciprofloxacin affects the ATPase activity of ABCB1 using the crude membranes of High-five insect cells. Our results showed that ciprofloxacin stimulates the ATPase activity of ABCB1 which is indicative of its interaction with the drug-substrate-binding site and competitive inhibition of ABCB1 activity.

Collectively, this is the first study to report the chemosensitizing effects of ciprofloxacin. Ciprofloxacin, at non-toxic concentrations, reverses ABCB1-mediated MDR by inhibiting its efflux function. However, the exact interaction of ciprofloxacin with the ABCB1 transporter is yet to be explored in in vivo models of MDR.

## 4. Material and Methods

### 4.1. Reagents

Ciprofloxacin was obtained from Tocris Bioscience (Minneapolis, MN, USA). Dulbecco’s modified Eagle’s Medium (DMEM), fetal bovine serum (FBS), penicillin/streptomycin and trypsin 0.25% were purchased from Hyclone (Pittsburgh, PA, USA). Monoclonal antibody against ABCB1 and GAPDH were purchased from Thermo Fisher Scientific Inc. (Rockford, IL, USA). Paclitaxel, vincristine, cisplatin, and verapamil were purchased from Sigma-Aldrich (St. Louis, MO, USA). [^3^H]-paclitaxel (15 Ci/mmol) was purchased from Moravek Biochemicals, Inc (Brea, CA, USA). The chemicals for the ATPase assay were same as those in our previous study [11].

### 4.2. Cell Lines and Cell Culture

The human colorectal adenocarcinoma cell line SW620, its doxorubicin-selected SW620/AD300 cell line, HEK293/pcDNA3.1, and HEK293/ABCB1 were used for ABCB1 reversal study. The HEK293/pcDNA3.1 and HEK293/ABCB1 cells lines were established by transfecting HEK293 cells with either the empty pcDNA3.1 vector or the vector containing full length ABCB1 (HEK293/ABCB1) DNA, respectively, and were cultured in a medium containing 2 mg/mL of G418. All cell lines were cultured at 37 °C, using 5% CO2 with DMEM containing 10% FBS and 1% penicillin/streptomycin. All drug-resistant cell lines were grown as adherent monolayer in a drug-free culture media for more than 2 weeks prior to their use.

### 4.3. MTT Assay

The modified MTT colorimetric assay was used to detect the sensitivity of cells to anticancer drugs in vitro as previously described [13,17]. Briefly, the parental and resistant cells were trypsinized, resuspended, and seeded into 96 well plates. After 24 h incubation, ciprofloxacin at the indicated concentrations (0–100 μM) was added to determine its cytotoxicity. To determine the reversal effects of ciprofloxacin, different concentrations of substrate chemotherapeutic drugs (20 μL/well) were added after pre-incubation with ciprofloxacin or verapamil for 2 h. After 72 h of incubation, MTT reagent (4 mg/ml and 20 μL/ well) was added and the plates were further incubated for 4 h. Subsequently, the MTT and medium was removed and 100 μL DMSO was added to dissolve the formazan crystals formed by the viable cells. Absorbance was determined at 570 nm by Opsys microplate reader (Dynex Technologies, Chantilly, VA, USA). The IC_50_ (concentration required to inhibit the growth by 50%) values were calculated from the survival curves using modified Bliss method [37]. Resistance fold (FR) was calculated by dividing the IC_50_ for the resistant cells with or without an inhibitor by that of the parental cells without an inhibitor. The concentrations of ciprofloxacin as a potential reversal agent used in this study were 1, 5, and 10 μM. Verapamil (10 µM) was used as a positive control inhibitor of ABCB1.

### 4.4. [^3^H]-Paclitaxel Accumulation Assay

The accumulation of [^3^H]-paclitaxel in the parental and resistant cells was measured in the presence or absence of ciprofloxacin and verapamil. Briefly, the cells were trypsinized and incubated in DMEM containing ciprofloxacin (1, 5, and 10 μM) and verapamil (10 µM) at 37 °C for 2 h. Cells were further incubated in DMEM containing 0.01 µM [^3^H]-paclitaxel with or without an inhibitor at 37 °C for 2 h. Subsequently, the cells were washed twice in ice cold PBS and lysed with 10 mM lysis buffer (pH 7.4, containing 1% Triton X−100 and 0.2% SDS). The lysed cells were placed in scintillation vials with 5 ml scintillation fluid and radioactivity was measured in a Packard TRI-CARB 1900CA liquid scintillation analyzer from Packard Instrument Company, Inc (Downers Grove, IL, USA).

### 4.5. [^3^H]-Paclitaxel Efflux Assay

In order to determine the efflux of [^3^H]- paclitaxel from the parental and resistant cells, the cells were incubated with 0.01 µM [^3^H]- paclitaxel as described in the accumulation experiment. After washing two times with ice cold PBS, the cells were incubated in fresh DMEM at 37 °C with or without an inhibitor. Aliquots of cell suspension were taken at 0, 30, 60, and 120 min and washed twice with ice cold PBS. Subsequently, the cells were lysed with 10 mM lysis buffer (pH 7.4, containing 1% Triton X−100 and 0.2% SDS) and placed in scintillation vials with 5 ml scintillation fluid. Radioactivity was measured in a Packard TRI-CARB 1900CA liquid scintillation analyzer from Packard Instrument Company, Inc (Downers Grove, IL, USA).

### 4.6. Preparation of Total Cell Lysate

The ciprofloxacin-treated and control cells were harvested and washed with ice cold PBS three times. Cell lysates were prepared with lysis buffer (10 mM Tris HCl, pH 7.5, 1 mM EDTA, 0.1% SDS, 150 mM NaCl, 1% Triton X−100 and 0.01% leupeptin) for 30 min on ice, followed by centrifugation at 12,000 rpm at 4 °C for 20 min. The supernatant was collected and stored at −80 °C for the Western blot analysis. Protein concentration was determined by bicinchoninic acid (BCA™)-based protein assay (Thermo Scientific, Rockford, IL, USA).

### 4.7. Western Blotting

Equal amounts of total cell lysates (50 μg protein) were resolved by sodium dodecyl sulfate polyacrylamide gel electrophoresis (SDS-PAGE) and electrophoretically transferred onto polyvinylidene fluoride (PVDF) membranes. The PVDF membranes were blocked with 5% skim milk dissolved in TBST buffer (10 mmol·L^−1^ Tris-HCL, 150 mmol·L^−1^ NaCl and 0.1% Tween20 pH 8.0) to block non-specific binding for 2 h at room temperature. The blot was probed with primary antibody C219 (ABCB1 specific; dilution 1:500) and 13E5 (β-actin specific; dilution 1:1000), overnight at 4 °C, followed by incubation with HRP (horseradish peroxidase)-conjugated secondary antibody, on the next day. The enhanced chemiluminescence detection system (Amersham, NJ, USA) was used to detect the protein-antibody complex. The expression of β-actin was used as a loading control and the protein expression was quantified by ImageJ 1.47v Software (NIH, MD, USA).

### 4.8. ATPase Assay

The vanadate-sensitive ATPase activity of ABCB1 in crude membranes of High-five insect cells was measured in the presence of ciprofloxacin (0 to 40 μM) by PREDEASY ATPase Kits with modified protocols, as previously described [7,35].

### 4.9. Molecular Modeling

Docking experiments were performed on a Mac Pro 6-core Intel Xenon X5 processor with Macintosh Operating System (OS X El Capitan) using Schrodinger 2015 [38] (Schrödinger, LLC, New York, NY, USA, 2015) software. Ligand preparations for all of the compounds were done using Lig-prep. A homology model of human P-gp was used which was already available [39] Protein preparation of the homology model was performed using ‘Protein Preparation Wizard’. The grid was generated by selecting residues at 20 Å from bound inhibitors in the homology model proteins (template PDBs: 4Q9I, 4Q9J, 4Q9K, 4Q9L) [38] The residues chosen were: 61, 64, 65, 68, 69, 72, 118, 125, 222, 299, 303, 306, 307, 310, 336, 339, 340, 342, 343, 721, 725, 728, 729, 732, 770, 841, 842, 870, 871, 872, 942, 945, 949, 953, 957, 975, 978, 979, 982, 983, 984, 985, 986, 987, 990, and 991. Extra Precision docking was performed with maximum 10 poses [40].

### 4.10. Statistical Analysis

All experiments were repeated at least three times and the differences were determined using the two-tailed student’s *t*-test (Microsoft Excel 2010) and statistical significance was determined at *p* < 0.05.

## Figures and Tables

**Table 1:** The effect of ciprofloxacin on reversal of ABCB1-mediated MDR.

Compounds	SW620		SW620/AD300	
	IC_50_ ± SD ^a^ (μM)	FR ^b^	IC_50_ ± SD ^a^ (μM)	FR ^b^
Paclitaxel	0.08 ± 0.01	[1.0]	27.38 ± 2.51	[342.2]
+ Ciprofloxacin 1 μM	0.09 ± 0.02	[1.1]	19.58 ± 1.86	[244.7]
+ Ciprofloxacin 5 μM	0.08 ± 0.01	[1.0]	5.71 ± 0.48	[71.3] ^c^
+ Ciprofloxacin 10 μM	0.07 ± 0.01	[0.8]	0.81 ± 0.03	[10.1] ^c^
+ Verapamil 10 μM	0.06 ± 0.01	[0.7]	0.92 ± 0.04	[11.5] ^c^
Vincristine	0.07 ± 0.02	[1.0]	16.44 ± 1.22	[234.8]
+ Ciprofloxacin 1 μM	0.07 ± 0.01	[1.0]	10.21 ± 0.99	[145.8]
+ Ciprofloxacin 5 μM	0.06 ± 0.01	[0.8]	3.89 ± 0.24	[55.5] ^c^
+ Ciprofloxacin 10 μM	0.05 ± 0.01	[0.7]	0.63 ± 0.03	[9.0] ^c^
+ Verapamil 10 μM	0.06 ± 0.02	[0.8]	0.69 ± 0.04	[9.8] ^c^
Cisplatin	2.23 ± 0.08	[1.0]	2.49 ± 0.09	[1.1]
+ Ciprofloxacin 1 μM	2.04 ± 0.09	[0.9]	2.51 ± 0.12	[1.1]
+ Ciprofloxacin 5 μM	2.17 ± 0.13	[0.9]	2.39 ± 0.19	[1.0]
+ Ciprofloxacin 10 μM	2.54 ± 0.09	[1.1]	2.78 ± 0.15	[1.2]
+ Verapamil 10 μM	2.69 ± 0.11	[1.2]	2.84 ± 0.14	[1.2]

^a^ IC_50_ values are represented as mean ±SD of at least three independent experiments performed in triplicate.^b^ FR: Resistance fold was calculated by dividing the IC50 values of substrates in the presence or absence of inhibitor by the IC50 of parental cells without inhibitor. ^c^ *p* < 0.05 versus the control group without reversal agent.

**Table 2:** The effect of ciprofloxacin on reversal of ABCB1-mediated MDR.

Compounds	HEK293/pcDNA3.1		HEK/ABCB1	
	IC_50_ ± SD ^a^ (μM)	FR ^b^	IC_50_ ± SD ^a^ (μM)	FR ^b^
Paclitaxel	0.07 ± 0.01	[1.0]	3.48 ± 0.31	[49.7]
+ Ciprofloxacin 1 μM	0.06 ± 0.03	[0.8]	1.83 ± 0.12	[26.1]
+ Ciprofloxacin 5 μM	0.06 ± 0.02	[0.8]	0.72 ± 0.04	[10.2] ^c^
+ Ciprofloxacin 10 μM	0.05 ± 0.01	[0.7]	0.24 ± 0.03	[3.42] ^c^
+ Verapamil 10 μM	0.08 ± 0.01	[1.1]	0.22 ± 0.04	[3.14] ^c^
Vincristine	0.04 ± 0.01	[1.0]	1.51 ± 0.08	[37.7]
+ Ciprofloxacin 1 μM	0.05 ± 0.02	[1.2]	1.02 ± 0.06	[25.5]
+ Ciprofloxacin 5 μM	0.04 ± 0.01	[1]	0.63 ± 0.03	[15.7] ^c^
+ Ciprofloxacin 10 μM	0.04 ± 0.01	[1]	0.11 ± 0.03	[2.7] ^c^
+ Verapamil 10 μM	0.05 ± 0.02	[1.2]	0.09 ± 0.04	[2.2] ^c^
Cisplatin	1.38 ± 0.16	[1.0]	1.59 ± 0.08	[1.1]
+ Ciprofloxacin 1 μM	1.25 ± 0.09	[0.9]	1.29 ± 0.10	[0.9]
+ Ciprofloxacin 5 μM	1.18 ± 0.13	[0.8]	1.32 ± 0.11	[0.9]
+ Ciprofloxacin 10 μM	1.31 ± 0.22	[0.9]	1.44 ± 0.08	[1.0]
+ Verapamil 10 μM	1.42 ± 0.15	[1.0]	1.33 ± 0.09	[0.9]

^a^ IC_50_ values are represented as mean ±SD of at least three independent experiments performed in triplicate.^b^ FR: Resistance fold was calculated by dividing the IC50 values of substrates in the presence or absence of inhibitor by the IC50 of parental cells without inhibitor. ^c^ *p* < 0.05 versus the control group without reversal agent.
